# Resolution of Consumptive Hypothyroidism Secondary to Infantile Hepatic Hemangiomatosis with a Combination of Propranolol and Levothyroxine

**DOI:** 10.4274/jcrpe.4865

**Published:** 2018-07-31

**Authors:** Victoria Campbell, Rachel Beckett, Noina Abid, Susannah Hoey

**Affiliations:** 1 Royal Victoria Hospital, Clinic of Dermatology, Belfast, Northern Ireland, United Kingdom; 2 Royal Belfast Hospital for Sick Children, Clinic of Paediatric Endocrinology, Growth and Diabetes, Belfast, Northern Ireland, United Kingdom

**Keywords:** Hemangioma, consumptive hypothyroidism, type 3 iodothyronine deiodinase, propranolol

## Abstract

Infantile hepatic hemangiomas (IHH), particularly of the diffuse subtype can, in severe cases, be associated with hepatic and cardiac failure, compartment syndrome and consumptive hypothyroidism. Early recognition and treatment of these pathologies is paramount in order to minimise the risk of long-term sequelae.

We report an interesting case of a female infant who presented with systemic compromise, in the absence of large or obvious cutaneous infantile hemangiomas. Imaging identified innumerable hepatic hemangiomas, consistent with diffuse infantile hepatic hemangiomatosis. Subsequent to this, thyroid function tests confirmed an associated but comparatively rare form of hypothyroidism, known as consumptive hypothyroidism. Following joint consultation with dermatology and endocrinology she was promptly treated with oral propranolol and levothyroxine, with subsequent improvement in her clinical parameters.

This case reiterates the importance of aggressive investigation and management of consumptive hypothyroidism in any infant diagnosed with IHH, particularly when there is systemic compromise. We advocate propranolol as a single first line treatment for IHH, supported by thyroid replacement when appropriate.

## What is already known on this topic?

Cutaneous infantile hemangiomas can be associated with significant hepatic involvement. Diffuse hepatic hemangiomatosis is associated with a unique and challenging form of hypothyroidism known as consumptive hypothyroidism.

## What this study adds?

In cases of systemic compromise, infants with hepatic hemangiomatosis should be screened for hypothyroidism at an early stage, even in the absence of obvious cutaneous clues. We advocate propranolol as a single first line agent to treat diffuse infantile hepatic hemangioma with systemic decompensation. Coexisting consumptive hypothyroidism should be aggressively managed to prevent long-term intellectual and developmental sequelae.

## Introduction

Infantile hemangiomas (IH) are benign endothelial cell neoplasms and the most common tumours of infancy, occurring in 3-5% of infants (1). They are more common in preterm and low birth weight infants and have a distinct pattern of proliferation during the first year of life, followed by gradual involution (2,3). While most IH are cutaneous, extracutaneous involvement of the liver may also occur. Although histologically benign and frequently asymptomatic, infantile hepatic hemangioma (IHH) can manifest as congestive heart failure associated with vascular shunting, abdominal compartment syndrome and fulminant hepatic failure with consumptive hypothyroidism, leading to death in the most severe cases.

In 2007 Christison-Lagay et al (4) divided IHH into three groups-focal, multifocal, and diffuse-based on the pattern and extent of liver involvement, correlated with clinical risk and outcomes. Focal lesions are predominantly glucose transporter (GLUT)-1 negative, and since they are formed *in utero* are amenable to antenatal diagnosis using ultrasound. They often lack associated cutaneous lesions, and as such may be missed if the presence of cutaneous hemangiomas is the sole stimulus to screen for hepatic involvement (5).Focal lesions have the potential to regress rapidly, behaviour akin to cutaneous, rapidly involuting congenital hemangiomas. In contrast, multifocal hepatic lesions are typically associated with multiple, small cutaneous IH, and are GLUT-1 positive. Most remain asymptomatic, and spontaneously resolve without sequelae. A minority have been associated with congestive cardiac failure (6).

Diffuse hepatic hemangiomas are associated with the highest risk of morbidity and mortality, secondary to massive infiltration of the hepatic parenchyma with innumerable hemangiomas. The diffuse subtype is associated with the rare entity of consumptive hypothyroidism, first described by Huang et al (7) in 2000.

The three types of iodothyronine deiodinases that regulate thyroid hormone activity are classified as types 1, 2 and 3. Type 3 iodothyronine deiodinase (D3) is a selenoenzyme, normally present in brain, placenta and fetal liver, and works by catalysing the conversion of thyroxine (T4) to reverse triiodothyronine (rT3) and the conversion of triiodothyronine (T3) to 3,3’-diiodothyronine, both of which are biologically inactive. High levels of D3 have been reported in hemangioma tissue (7). Consumptive hypothyroidism is characterised by low free T3 (fT3) and normal or low free T4 (fT4), despite elevated thyroid stimulating hormone (TSH) (8,9). Patients have elevated serum rT3 levels as a result of increased T4 and T3 degradation by D3 (7,10).

In a review of 30 published cases of diffuse IHH, Yeh et al (6) reported that more than 70% were hypothyroid, with eleven requiring treatment. They postulated that hypothyroidism may have been occult in the remaining cases. Thyroid hormones are crucial for growth and neurodevelopment during early childhood, with three to five IQ points lost for each month in which hypothyroidism remains untreated in the first year of life (11).This developmentally critical period parallels the proliferative phase of hemangiomas and highlights a window of opportunity to screen for and aggressively treat hypothyroidism in the context of diffuse hepatic hemangiomas.

Here we report a female infant with diffuse IHH and consumptive hypothyroidism, successfully managed with propranolol and levothyroxine.

## Case Report

(Parental informed consent was obtained prior to writing and publication of this case, inclusive of images).

A female twin conceived through in vitro fertilization was born via normal vaginal delivery at 34+3 weeks to non-consanguineous parents, weighing 1.98 kg. The antenatal and perinatal periods were reported to be uneventful. Her older brother and twin are both well, and there was no relevant family history. The patient presented to our hospital with complaints of poor feeding and pallor at age eleven days. Her initial C-reactive protein (CRP) was elevated at 55 mg/L, and she was treated with antibiotics. She presented again at age three weeks in extremis with reduced consciousness, pallor, tachycardia, tachypnoea, epistaxis after feeding and abdominal distension. Petechiae were noted on her lower limbs. She was intubated and transferred to intensive care, where a chest X-ray suggested infection. She was again treated for possible sepsis with intravenous amoxicillin and cefotaxime. Ventilatory support was weaned and she was extubated after twenty-four hours. She received a unit of blood for anaemia (haemoglobin 6.4 g/dL prior to transfusion).

She was again readmitted at age eight weeks following an unresponsive episode, ongoing feeding difficulties with vomiting, and a distended, tense abdomen. On this occasion, an abdominal X-ray revealed hepatomegaly. Ultrasound of the abdomen showed innumerable hypoechoic nodules and increased vascularity within the liver, confirmed on computed tomography and magnetic resonance imaging. Alpha fetoprotein was markedly elevated at 1165 KU/L (normal range 0-10 KU/L), with associated derangement of her liver function tests and coagulation profile. High output cardiac failure was diagnosed, with a N-terminal pro-brain natriuretic level of 1492 ng/L (normal range <115 ng/L). Diuretics were commenced with good effect. A baseline echocardiogram indicated a mildly dilated left heart.

**Figure 1:**
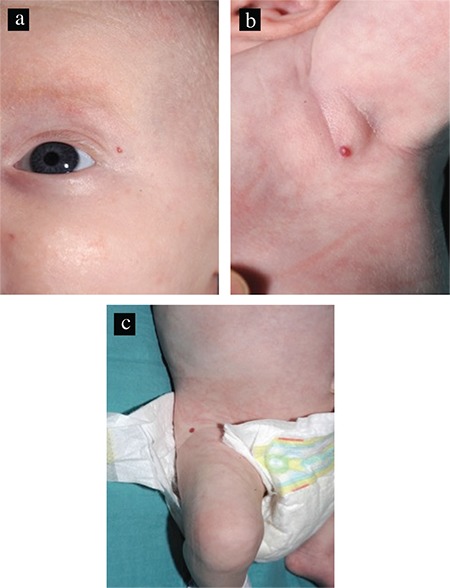
Cutaneous infantile hemangiomas a) at the left lateral canthus, b) left axilla, c) and right lateral thigh

Incidentally, a small (3 mm) cutaneous haemangioma at the right lateral thigh was noted during abdominal ultrasound. Following a dermatology review, two further small cutaneous hemangiomas were identified at the left lateral canthus and left axilla (Figure 1a, 1b and 1c).

**Figure 2:**
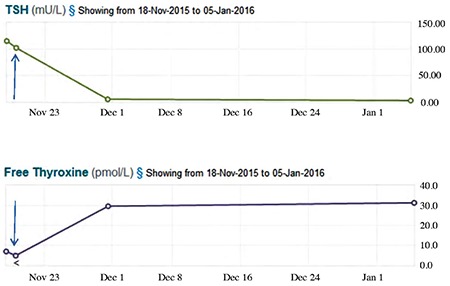
Trends in thyroid stimulating hormone and free thyroxine over time after treatment with levothyroxine at 9.6 micrograms/kg/day TSH: thyroid stimulating hormone

In view of the combined cutaneous and radiological findings, thyroid function tests were checked and found to be grossly abnormal, with an initial fT4 of 7.1 pmol/L (normal range 9-20 pmol/L) and a TSH of 115.4 mU/L (normal range 0.35-4.94 mU/L). They were repeated a day later, showing a fT4 of <5.0 pmol/L, a fT3 of 2.3 pmol/L (normal range 3.0-9.28 pmol/L) and a TSH of 102.5 mU/L, in keeping with consumptive hypothyroidism. Following consultation with the pediatric endocrinologists, levothyroxine at a dose of 9.6 µg/kg once daily was commenced. Eleven days later, TSH had normalised to 5.33 mU/L and fT4 was appropriately elevated at 29.6 pmol/L (Figure 2).

**Figure 3:**
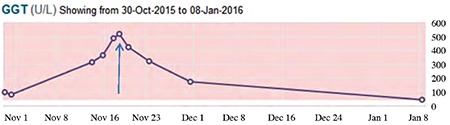
Trend in gamma-glutamyl transpeptidase over time. The arrow indicates when propranolol was commenced GGT: gamma-glutamyl transpeptidase

Following discussion with colleagues in dermatology and cardiology, the patient was started on propranolol 1 mg/kg once daily, in two divided doses (6,12,13,14,15). This was escalated to 2 mg/kg after five days, with close monitoring of blood pressure, heart rate and capillary glucose levels. Treatment was well tolerated with no documented side effects, and within two days of commencing propranolol gamma-glutamyl transpeptidase had decreased from 522 to 426 U/L (normal range 6-42 U/L) and continued to do so in a linear fashion (Figure 3). This coincided with clinical improvement and a subsequent ultrasound at eighteen weeks of age confirmed improvement in the hepatomegaly, with a reduction in the size and number of lesions. This correlated with involution of the cutaneous hemangiomas. Post-treatment the child is well, with normal developmental milestones.

## Discussion

The potential for consumptive hypothyroidism, hepatic and cardiac failure, and abdominal compartment syndrome prompted Dickie et al (16) to recommend that an abdominal ultrasound should be obtained to assess for IHH in any infant (symptomatic or asymptomatic) younger than six months of age who presents with five or more cutaneous IH. This recommendation is in line with Horii et al (5), who confirmed the trend for a greater risk of IHH with increasing numbers of cutaneous IH. However, it has also been reported that IHH can cause liver disease in the absence of any cutaneous lesions and this case, where only three small and easily missed cutaneous IH were identified, highlights the importance of having a low threshold to perform abdominal ultrasound in a child with any cutaneous lesions and systemic compromise (16). The signs of systemic compromise may be subtle, and include failure to thrive (secondary to underlying thyroid or cardiac dysfunction) and feeding difficulties. The presence of hepatomegaly on clinical examination should expedite radiological investigation.

Yeh et al (6) recognised that cutaneous IH are heterogeneous in morphology, varying from small papules to large segmental areas of involvement. In their case series of four infants with diffuse IHH, all the cutaneous IH were firm, thick, dome-shaped nodules. They recognised that further reports on the morphology of cutaneous IH in the setting of diffuse IHH would be of interest to determine if this could be used as a predictor for the diffuse pattern of hepatic disease (6). Again, our case emphasises that not just the number, but also the morphology of cutaneous IH cannot always be reliably used as an indicator of internal and systemic involvement.

The importance of consumptive hypothyroidism as a diagnosis mandates screening for thyroid abnormalities in those infants with identified IHH, particularly those with the diffuse subtype (5,6). Consultation with endocrinology for prompt and specialist management of hypothyroidism is imperative if growth and irreversible intellectual retardation are to be prevented (6,10). In 2000, a report on severe hypothyroidism in the context of IHH suggested that “given the adaptive capacity of the thyroid gland, it is likely that only patients with both high levels of D3 activity and large tumour burdens are at risk for hypothyroidism” (7).This statement underpins the rationale as to why consumptive hypothyroidism is most prevalent in the diffuse subtype of IHH. The aetiology of elevated D3 in IHH is not fully understood, but some postulate that it is due to similarities between the endothelial cells in hemangiomas and those in placenta, which share certain immunohistochemical markers such as GLUT-1. Furthermore, it has been proposed that IH could be derived from placental angioblasts, and would explain the placenta-like characteristics of IHH such as high D3 activity and self-limited growth (10,17,18). Whatever the cause of consumptive hypothyroidism, clinicians must be mindful of the sometimes recalcitrant nature of this specific form of hypothyroidism and be willing to quickly escalate to higher than usual doses of levothyroxine and/or liothyronine in order to minimise the risk of long term sequelae. The dose required varies on an individual basis; in this case there was a rapid and sustained response to a dose of 9.6 µg/kg levothyroxine once daily (equivalent to a total dose of 37.5 µg/day). Much higher doses have been reported in the literature, with Emir et al (19) reporting the use of levothyroxine 75 µg/day in a female infant with IHH and associated consumptive hypothyroidism, and most recently Al Tasseh et al (20) documenting a dose of levothyroxine 25 µg/kg/day in order to achieve a euthyroid state in a 3.5-month-old male with diffuse IHH.

Although a percentage of patients with IHH may experience spontaneous regression, the development of systemic and life-threatening complications merits prompt treatment (21). Propranolol (a nonselective beta-blocker) has evolved to become a well accepted treatment option for cutaneous IH since its serendipitous discovery in 2008 (22). Propranolol has the combined advantage of promoting more rapid involution of the hemangioma, in addition to halting its growth.

More recently, there has been a growing body of evidence suggesting the benefit of propranolol in IHH (6,12,13,15).Traditional treatments for IHH have included systemic steroids, interferon and vincristine, all of which are associated with potentially severe or dangerous side effects (15). Conversely, propranolol is regarded as a well-tolerated treatment with a favourable risk-benefit ratio. In a case series of eight infants with IHH and diffuse neonatal hemangiomatosis, Mazereeuw-Hautier et al (14) reported rapid and dramatic efficacy of propranolol in all cases, both in the presence and absence of heart failure, and irrespective of whether it was used as a single agent or in combination with other therapies. No side effects of the drug were reported. These findings led them to conclude that propranolol is a valid first line treatment for IHH.

Yeh et al(6) were more cautious in their recommendations having reported four cases of diffuse IHH, and advocated early treatment with combined corticosteroids and propranolol, whilst acknowledging that the use of propranolol in infants with symptomatic IHH merits further study to elucidate if propranolol alone or in combination with steroids is most optimal.

Here we report a further case of diffuse IHH successfully managed with propranolol as a single, first line agent, well tolerated and with no adverse effects. Early recognition of coexisting consumptive hypothyroidism and cardiac failure, coupled with careful dermatological examination in the absence of obvious or numerous cutaneous clues, resulted in prompt involvement of the relevant specialties and timely treatment. More research is needed to fully understand the pathophysiology underlying systemic decompensation in diffuse IHH and to understand the exact mechanism of action of propranolol when used as a first line treatment in this context.
